# An Analysis of the Influence of Flight Parameters in the Generation of Unmanned Aerial Vehicle (UAV) Orthomosaicks to Survey Archaeological Areas

**DOI:** 10.3390/s16111838

**Published:** 2016-11-01

**Authors:** Francisco-Javier Mesas-Carrascosa, María Dolores Notario García, Jose Emilio Meroño de Larriva, Alfonso García-Ferrer

**Affiliations:** Department of Graphic Engineering and Geomatics, University of Cordoba, Campus de Rabanales, Córdoba 14071, Spain; maranotario@gmail.com (M.D.N.G.); jemerono@uco.es (J.E.M.d.L.); agferrer@uco.es (A.G.-F.)

**Keywords:** unmanned aerial vehicle (UAV), positional quality, orthomosaick, archeology

## Abstract

This article describes the configuration and technical specifications of a multi-rotor unmanned aerial vehicle (UAV) using a red–green–blue (RGB) sensor for the acquisition of images needed for the production of orthomosaics to be used in archaeological applications. Several flight missions were programmed as follows: flight altitudes at 30, 40, 50, 60, 70 and 80 m above ground level; two forward and side overlap settings (80%–50% and 70%–40%); and the use, or lack thereof, of ground control points. These settings were chosen to analyze their influence on the spatial quality of orthomosaicked images processed by Inpho UASMaster (Trimble, CA, USA). Changes in illumination over the study area, its impact on flight duration, and how it relates to these settings is also considered. The combined effect of these parameters on spatial quality is presented as well, defining a ratio between ground sample distance of UAV images and expected root mean square of a UAV orthomosaick. The results indicate that a balance between all the proposed parameters is useful for optimizing mission planning and image processing, altitude above ground level (AGL) being main parameter because of its influence on root mean square error (RMSE).

## 1. Introduction

Traditional archaeological site surveys are a time-consuming effort proportional to the difficulty of access of the site being investigated. Prospective sites are identified based on oral tradition, written records or inspection using images [1]. Once a prospective site is identified, fieldwork starts to detect evidence of human activity. With images, these have usually been registered by sensors on board two traditional platforms, satellite and manned aircraft [2,3,4]. These platforms present problems with spatial resolution applied to archaeology. One of the limiting factors of satellite images is the difficulty to detect small- or even medium-sized details like sites smaller than a hectare [3]. There are also reasons to be cautious about the effectiveness of satellite imagery in detecting prehistoric sites lacking a long history of occupation [5]. On the other hand, manned aircraft are able to supply images with better spatial resolution but no higher than at a 1:500 scale. Moreover, the economical cost of aerial photogrammetry is usually too high for small surveyed areas [6].

Currently, unmanned aerial vehicles (UAVs) are an alternative for the acquisition of images with a very high spatial resolution for documenting archeological areas [7]. UAVs are classified with different characteristics like range, endurance, mass and architecture. Generally, most common UAV categories used in civil applications are micro and mini UAVs with a mass of less than 5 and 150 kg, respectively. Other characteristics, like endurance or range, depend mainly on the type of platform architecture, for example multirotor, fixed wing and balloon.

Different types of UAVs have been used successfully to survey archeological areas such as helium balloons [8], blimp [9], kites [10], fixed wing [11] and rotor wing [12]. In mapping, UAV flight parameters are critical in obtaining adequate spatial quality on the derived geomatic products to survey archeological sites. The correlation between flight parameters, spatial quality and photogrammetric processing of images acquired by metric sensors have been well studied in classic photogrammetry [13]. Accuracy assessment of digital elevation models [14] or the influence of Ground Control Points (GCPs) in aerial-triangulation [15], among others, have contributed to defining a standardized processing framework.

In contrast, UAV photogrammetry for research applications is still at an early stage [16]. One consequence of this is that operational frameworks for working with UAV platforms are not defined in some aspects and applications. The operational framework depends on the type of UAV platform, sensors on board and case of use. Ref. [17] analyzes the potential of UAVs for measuring area of land plots for monitoring land policies, Ref. [18] explored the positional quality of orthophotos obtained by a UAV following the requirements of National Mapping Agencies, and Ref. [19] defines specifications to acquire remote images using a six-band multispectral sensor on board a UAV for use in precision agriculture. Regarding cultural heritage, UAVs have rarely been used in scientific research [20]. Parameters like altitude above ground level (AGL), number of GCPs, or the percentage of forward-lap and side-lap determine the spatial quality of orthomosaics.

One of the most important parameters in an UAV flight is altitude AGL. It determines the pixel size on the registered images, flight duration and area covered. Firstly, it is necessary to define the spatial quality requirements for the orthomosaics to achieve the ideal pixel size in the images registered by the sensor. In general, at least four pixels are required to detect the smallest detail in an image [21]. In selecting altitude AGL, sufficiently fine spatial resolution has to be guaranteed and, as the same time, as much surface as possible has to be covered. Very low altitude AGL UAV flights generate very high spatial resolution images but cover a limited area and therefore increase flight duration. As a result, the UAV operation has to be fragmented into different flights due to battery life, causing variations in illumination, the appearance or disappearance of shadows, saturated images, depending on the type of materials present, and so on.

As in traditional photogrammetry, the algorithms used process overlapping images acquired from multiple viewpoints. Mainly, these techniques are based on imaging techniques called structure from motion (SfM) [22]. SfM photogrammetry differs from conventional photogrammetric approaches by calculating internal camera parameters (focal length, principal point and distortion coefficients), camera position and orientation. SfM algorithms need a large number of overlapping images to cover the area of interest [23,24], which impacts flight duration. High percentages of forward and side overlap increase flight duration because it is necessary to capture more images for each individual lap and to increase the number of total laps. However, this improves the spatial quality of geomatic products. All of these parameters affect battery life and thus a balance between spatial quality, forward and side overlap and flight time duration is necessary.

Finally, GCP distribution and its influence on the spatial quality of orthomosaics in traditional platforms is well described [25]. With UAVs, the number and distribution of GCPs are not standardized, being analyzed by [26,27]. The consequence is that the number of GCPs may covers a broad range, from just four GCPs to more than 100 GCPs [27,28]. In addition, Ref. [29] analyzes the accuracy of UAV orthomosaics without GCPs, with the resulting root mean square error (RMSE) being higher than one meter.

To our knowledge, no detailed investigation has been conducted regarding the influence of UAV flight parameters such as altitude AGL, forward and side overlap, and the use or lack of GCPs on the spatial quality of orthomosaics using a red–green–blue (RGB) on board a multi-rotor UAV to be used in surveying archaeological areas. This paper defines the technical specifications for working with a multi-rotor UAV to obtain an accurate spatial orthomosaics to be used to survey archeological areas.

The manuscript is divided in the following sections: in Section 2, the technology, study area, and the materials and methods are described. In Section 3, results are presented, followed by conclusions in Section 4.

## 2. Materials and Methods

### 2.1. UAV and Sensor Description

**Figure 1:**
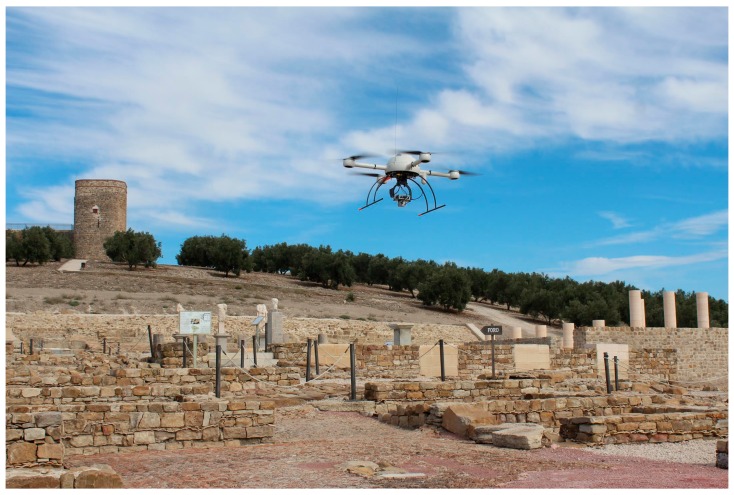
The MD4-1000 multi-rotor (Microdrones GmbH, Siegen, Germany) taking off over the study site.

The unmanned aerial vehicle used for mapping was a MD4-1000 multi-rotor (Microdrones GmbH, Siegen, Germany) (Figure 1). This UAV is a vertical takeoff and landing aircraft of an entirely carbon design. The system has a maximum payload of 1.2 kg. It uses 4 × 250 W gearless brushless motors powered by a 22.2 V battery. It reaches a cruising speed of 12.0 m/s and a maximum climb speed of 7.5 m/s. Its maximum wind tolerance is up to 12.0 m/s, registering steady picture up to 6 m/s. Flight duration depends on sensor weight and weather conditions. For this project, the multi-rotor UAV was equipped with a Sony NEX-7 RGB sensor (Sony Corporation, Minato, Tokyo, Japan) with a 16 mm lens. The camera weighs 353 g including the camera body, card and battery and provides a 23.5 × 15.6 mm image (6000 × 4000 pixels). The sensor was field calibrated and the results used in this research are summarized in Table 1. With this sensor, the UAV’s flight duration is approximately 30 min. During the flight, the sensor registers vertical images. The image trigger is activated by the UAV’s autopilot flight settings. For each shot, the UAV autopilot sends a signal to the sensor to register an image and simultaneously timestamps and records the GPS location and navigation angles (yaw, roll and pitch) on a Secure Digital Card (SD-Card). This information will be used for the initial values in photogrammetric processing.

### 2.2. Study Site and UAV Flights

**Figure 2:**
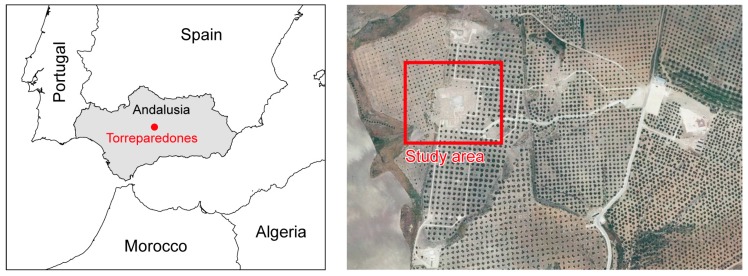
Overview of the study site.

The study area was approximately 1.13 ha (101 × 112 m) in size and was conducted in Torreparedones, an old Iberian and Roman town situated between the Guadalquivir river and the Guadajoz river in the province of Córdoba (Southern Spain) (37°45′ N, 4°22′ W) (Figure 2). This settlement has been continuously populated between the 2nd Century Before Christ (BC) and the 16th Century Anno Domini (AD). It reached its peak during the Iberian and Roman period as a municipality and played an important role in the commercial trade routes throughout the southern and eastern territories of the Peninsula as well as in the development of metallurgy. Evidence of public buildings like shrines and a forum have been found in the archaeological remains.

**Figure 3:**
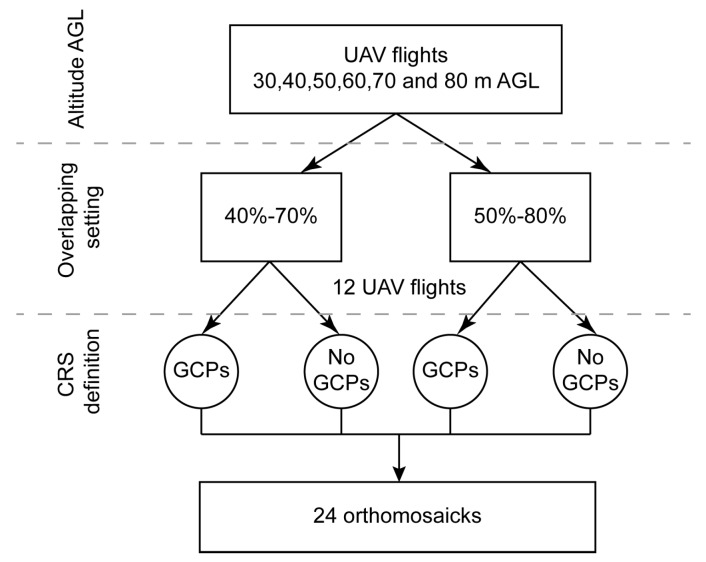
Scheme of unmanned aerial vehicle (UAV) flights and processing.

Several flights above the remains were planned following the scheme presented below in Figure 3, which combines different flight altitudes, overlap settings and the use, or lack thereof, of GCPs. Descriptions of flight parameters and their formula are widely available, for example [30].

One of the most important flight parameters is altitude AGL. In this study, a set of flight missions were flown at altitudes of 30, 40, 50, 60, 70 and 80 m AGL. Each altitude AGL is linked to a specific ground sample distance (GSD) value. In this study, GSD ranged from 0.7 cm × pixel^−1^ at 30 m AGL, to 2.0 cm × pixel^−1^ at 80 m AGL. Additionally, two different forward-lap and side-lap settings were used: 80%–50% and 70%–40%. In combining these settings, twelve missions were flown in total. All UAV flights were carried out under the same wind conditions, the wind speed being equal to 2 m/s. In addition, all UAV flights were planned in such a way that each point in the study area was captured in at least 3 images. Thus, the accuracy of the orthomosaicks was only dependent on altitude AGL and forward and side lap settings.

Afterwards, each UAV flight was processed with and without GCPs. In the former case, GCPs and the georreferentiation information registered by the UAV’s autopilot were used in the aerial triangulation phase to accurately place the photogrammetric block into a coordinate reference system (CRS). In the latter case, the aerial triangulation was processed with the information registered by the UAV’s autopilot, and nothing more. In using, or not using, GCPs with the information from the 12 flights, a total of 24 orthomosaics were produced to assess spatial quality.

**Figure 4:**
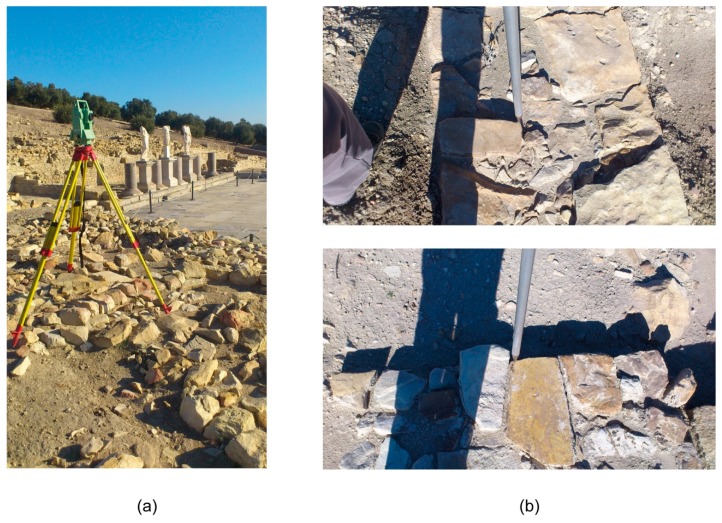
Assessing spatial resolution: (**a**) Measuring using the total station; and (**b**) Samples of spatial details of the ground measurements.

The coordinates of each GCP in the study area were determined by using traditional topography methodologies instead of global navigation satellite system (GNSS) sensors. This decision was made because the precision and accuracy of GCP coordinates have to be greater than the GSD of UAV flights. In this context, a GNSS sensor receiving real-time corrections does not obtain results greater than 2 cm. This value is higher or equal to the GSD of UAV flights, and, therefore, GNSS was rejected. GCPs were chosen in the corners of the study area, one for each corner, and another in the center. Each GCP was set with an artificial target and measured using a total station Leica TC805 (Leica Geosystem AG, Heerbrugg, Switzerland) (Figure 4a) with an angle accuracy equal to 5′′ and a distance measurement precision equal to ±(3 mm + 2 ppm).

### 2.3. Photogrammetric Processing

The photogrammetric processing is divided into 4 phases: (1) aerial triangulation; (2) Digital Surface Model (DSM) generation; (3) rectification of individual images; and, lastly (4) orthomosaicking.

Aerial triangulation is the basic method for analyzing aerial images in order to calculate the three-dimensional coordinates of object points and the exterior orientation of the images [31]. This process allows the absolute orientation of the entire photogrammetric block to be calculated. To perform the bundle adjustment, algorithms based on “Structure from Motion” (SfM) techniques are used. SfM algorithms operate with the same basic fundamentals of stereoscopic photogrammetry. However, it differs from traditional photogrammetry in the geometry of the scene, camera positions and orientation. Using UAV platforms, this is resolved using highly redundant information extracted from a set of multiple high percentage overlaps that register the three-dimensional structure of the scene [32]. In a first stage, SfM techniques extract individual features in each image of the photogrammetric block, which are afterward matched to their corresponding feature in the other images of the photogrammetric block. These features are used to determine the relative position of the sensor during the flight, which allows the position and orientation for each individual sensor to be calculated. At this stage, the spatial quality of the results depends on the quality of the geolocation sensor, GNSS sensor and IMU sensor. In general, the attributed geolocational accuracy of images taken on commercial UAVs is medium to low. Therefore, in this study, geolocation was calculated via aerial-triangulation. To improve the spatial quality of the results, a group of GCPs were distributed over the study area. These GCPs were measured on field with a greater spatial accuracy than GSD.

Once aerial-triangulation was calculated, a DSM was generated in three stages: feature extraction, multi-image matching and blunder detection [6]. DSM and external orientation were used to orthorectify each image. Finally, individual orthorectified images were mosaicked to obtain an UAV orthomosaic of the entire study area. Each orthomosaic was produced with a GSD equal to the corresponding GSD of each UAV flight.

The photogrammetric processing was performed using Inpho UASMaster (Trimble, CA, USA) [33].

### 2.4. Assessment of Spatial Quality

Spatial accuracy is the accuracy of the position of a feature related to Earth [34] and can be described in absolute or relative terms. Absolute accuracy is defined as the closeness of reported coordinate values to values accepted as or being true. Relative accuracy is defined as the closeness of the relative spatial positions of features in a dataset to their respective relative spatial positions accepted as or being true.

Before the UAV flights, 150 check points were measured in the study area to assess the absolute and relative spatial accuracies (Figure 4b). The check points’ coordinates were obtained using a total station in the same manner as the GCPs (Figure 4a) and were well-defined and well distributed. These coordinates were used as ground reference values to assess the spatial quality of the orthomosaics.

All check point locations were digitized on screen via the produced orthomosaics. These coordinates were obtained using Quantum GIS (QGIS) [35]. Both sets of ground and orthomosaic coordinates were compared to determine the spatial quality.

Absolute positional accuracy was assessed by RMSE, which is used to estimate positional accuracy [36]. Relative positional accuracy was assessed using the methodology developed by the Department of Defense of the United States [37]. Subsequently, all possible check point pair combinations were determined. Afterwards, the absolute and relative errors in the *X* and *Y* dimensions of each check point were calculated. These errors were used to calculate both the relative standard deviations on each axis and the relative horizontal standard deviation (RHSD).

## 3. Results

**Figure 5:**
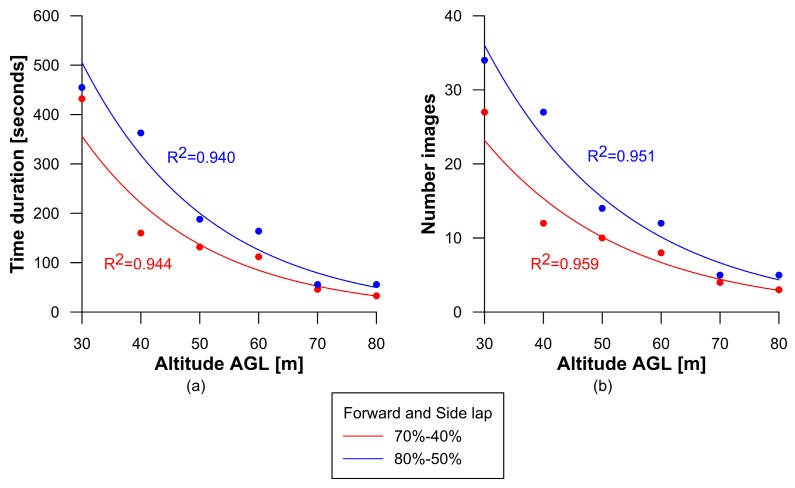
Relationship between altitude above ground level (AGL) and forward and side lap settings on: (**a**) Flight duration and (**b**) Number of images taken.

A total of two single flights missions were flown, one for each forward and side lap setting. Once the UAV completed the initial altitude, it ascended 10 m and flew the same area again. This process repeated until all programmed altitudes were covered. Table 2 summarizes the duration of, and number of images taken from, each UAV flight with each flight having a different altitude AGL and forward and side overlap setting. In Table 2, time duration expresses the duration of the flight for an individual altitude AGL, without time spent taking off and landing. Table 2 demonstrates, as altitude AGL increases, flight duration and the number of images taken as a decrease because each image covers more area, and, therefore, fewer laps and images are needed. This occurs independently of the forward and side overlap settings, although higher percentages increase flight time and number of images taken. The longest UAV flight was 7 min and 35 s at 30 m AGL with 80%–50% forward and side overlap, while the shortest was 33 s at 80 m AGL with 70%–40% forward and side overlap. Figure 5 shows an exponential correlation between altitude AGL, flight duration (Figure 5a) and the number of images taken (Figure 5b). Inversely, as altitude AGL was reduced, flight duration and the number of images taken exponentially increased.

The shape of the study area in relation to the percentage of forward and side overlap also affects the duration of UAV flights. In this study, there was a significant time difference at 40 m AGL due to the forward and side overlap settings. Fewer laps were needed to cover the area of study at 70%–40% because more distance needed to be covered between laps at 80%–50%. At higher altitudes AGL, for example 70 or 80 m, the differences in flight duration were reduced due to only one lap being needed to capture both forward and side overlap, which reduced the number of images taken.

Another factor that affects flight duration is illumination. Sometimes, elements may appear in the study area which can produce shadows depending on the direction of the flight. Moreover, some materials, like marble, reflect light intensely, resulting in highly saturated images. Consequently, flying within limited timeframes may be necessary to avoid problems caused by illumination. Therefore, reducing flight times while simultaneously maintaining orthomosaic spatial quality is of interest.

**Figure 6:**
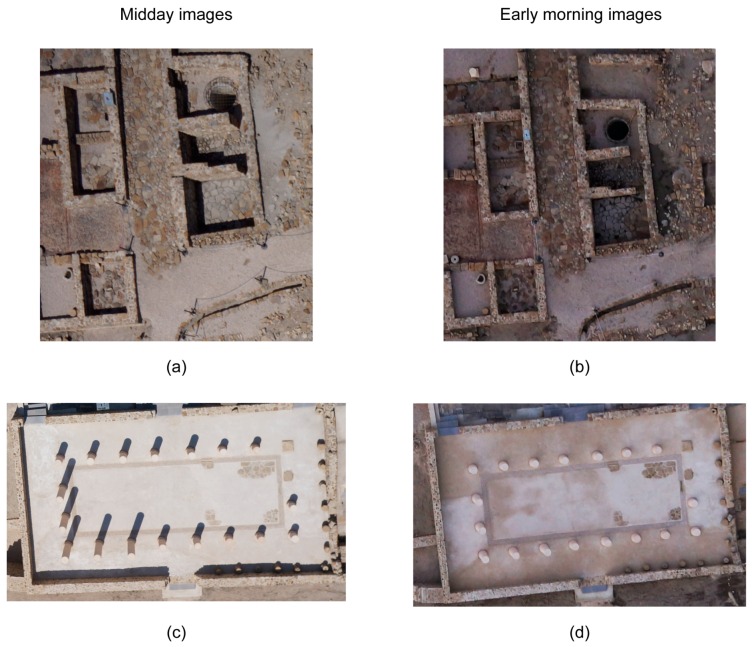
(**a**–**d**) The effects of illumination on images taken by UAV flights at midday (**a**,**c**) and early morning (**b**,**d**).

Figure 6 compares images taken in the early morning (7:15 a.m.) and again close to midday (11:30 a.m.) at the same altitude AGL. Elements such as walls (Figure 6a,b) or columns (Figure 6c,d) project shadows if the images are taken at midday (Figure 6a,c). On the other hand, if taken in the early morning, images do not contain shadows (Figure 6b,d). Because sun elevation is reduced and objects do not drop shadows, as such images are darker. One option, to avoid shadows, is to fly when the Sun is at the zenith position. In this case, it is necessary to take into account the coordinates of study area and day of the year to know when the Sun is at this position. However, in this case, problems can arise with materials like marble, which can saturate images leading difficulties in visual interpretation. As an example, in Figure 6c, it is more difficult to identify individual elements at the top of the wall compared to Figure 6d.

**Figure 7:**
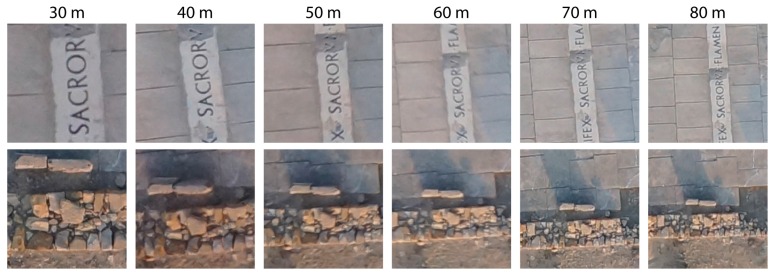
Effect of flight altitudes AGL on image coverage and quality.

Figure 7 shows an example of two sets of images of two different areas taken at different altitudes AGL and demonstrates that, as altitude AGL increases, the area covered by each image increases, reducing flight duration. On the other hand, the quality of spatial resolution and border definition of individual elements improve as altitude AGL decreases. At 30 m, element boundaries are well defined and recognizable in an individual context, while at higher altitude AGL, definition incrementally diffuses, and it becomes more difficult to define individual elements. However, this does not mean that UAV flights at high altitude AGL are not useful in archaeological utilities, and it depends on the need of the user. The geomatic product requirements of an archaeological area needing only a general map, which typically meets or exceeds user expectations, are not the same as those of a specific site prospection. Therefore, product features are said to posses “fitness for use” if they are able to serve their purposes [38].

From the point of view of image interpretation, altitudes equal to 80 m AGL or higher allow valuable information to be gained for a general analysis of the entire work area and surroundings although details less than 2 cm in size are more difficult to properly identify. Conversely, lower altitude AGL flights allow details to be better studied at the cost of increased flight duration.

### 3.1. Assesement of Absolute Positional Accuracy

**Figure 8:**
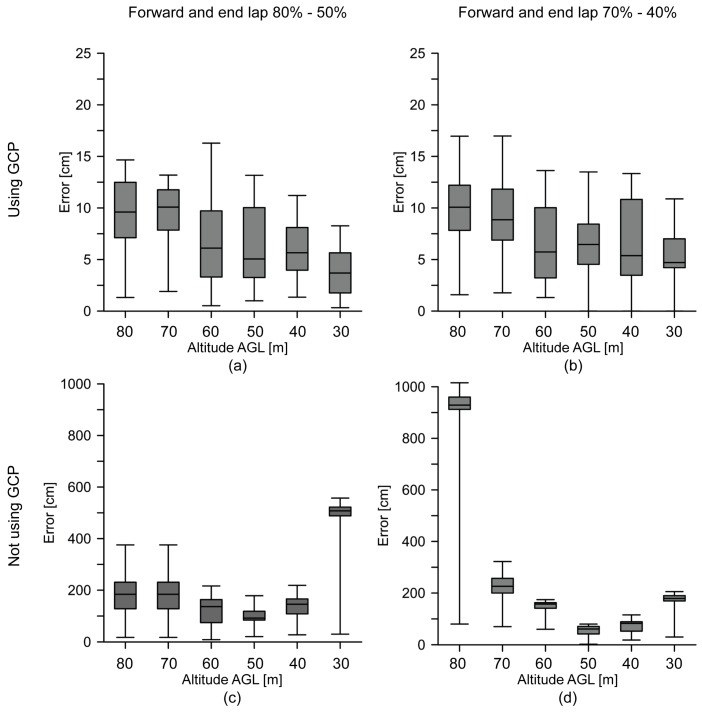
(**a**–**d**) Root mean square error (RMSE) box plot graph factoring altitude AGL; forward and side lap (**a**,**c**) 80%–50% (**b**,**d**) 70%–40% and; processing (**a**,**b**) with or (**c**,**d**) without ground control points (GCPs).

Table 3 summarizes the results of the absolute positional accuracy assessment factoring: (1) altitude AGL; (2) forward and side lap settings; and (3) processing with and without GCPs. Error ranges from 3.8 cm (30 m altitude AGL and 80%–50% overlap settings with GCPs) to 934.2 cm (80 m altitude AGL and 70%–40% overlap settings without GCPs). In Figure 8, the same factors from Table 3 are applied to error box plots. Figure 8a,b show a lower RMSE where GCPs were used than Figure 8c,d where GCPs were not used, these results being independent of altitude AGL and forward and side lap setting.

The absolute positional accuracy of orthomosaics produced without GCPs depends on the accuracy of the navigation system of the UAV. Currently, these systems generally have an accuracy of about 1 to 2 m, which is not accurate enough for direct georeferencing. Therefore, GCPs are necessary to properly define the coordinate reference system. Alternatively, integrating an accurate direct georeferencing system onto a UAV platform would allow the elimination of GCPs [39]. Although most commercial UAVs are not equipped with an accurate direct georeferencing system, there are UAVs in the market with this capability, which will likely be a more common solution in the future.

As altitude AGL increased, errors also increased when GCPs were used (Figure 8a,b). This behavior was constant independent of forward and side overlap settings. From 30 m to 40 m AGL, flights showed a positional accuracy of less than 5 cm. From 50 m to 60 m AGL, the RMSE was around 6 cm. RMSE was higher than 9 cm with altitudes 70 m AGL and up. This suggests that, as the altitude AGL increases, GSD of images increases, which is reflected in RMSE. On the other hand, the orthomosaics where GCPs were not used (Figure 8c,d) showed random RMSE behavior due to the lack of geometric constraints of calculating aerial-triangulation which was because the RMSE depended on the accuracy of the UAV’s navigational system, suggesting lower altitude AGL flights and the use of GCPs give better absolute positional accuracy.

With the forward and side overlap settings, higher percentages (Figure 8a) resulted in lower RMSE for all UAV flights. The cause of this may be that there was more redundant information to extract tie points. SfM algorithms applied to UAV flights show better results when using a high redundant bundle adjustment based on matching features in multiple overlapping images. As in [40], all individual flights with a forward and side lap equal to 80%–50%, respectively, showed better results than a 70%–40% configuration. These improved results were more evident at lower altitudes AGL.

**Figure 9:**
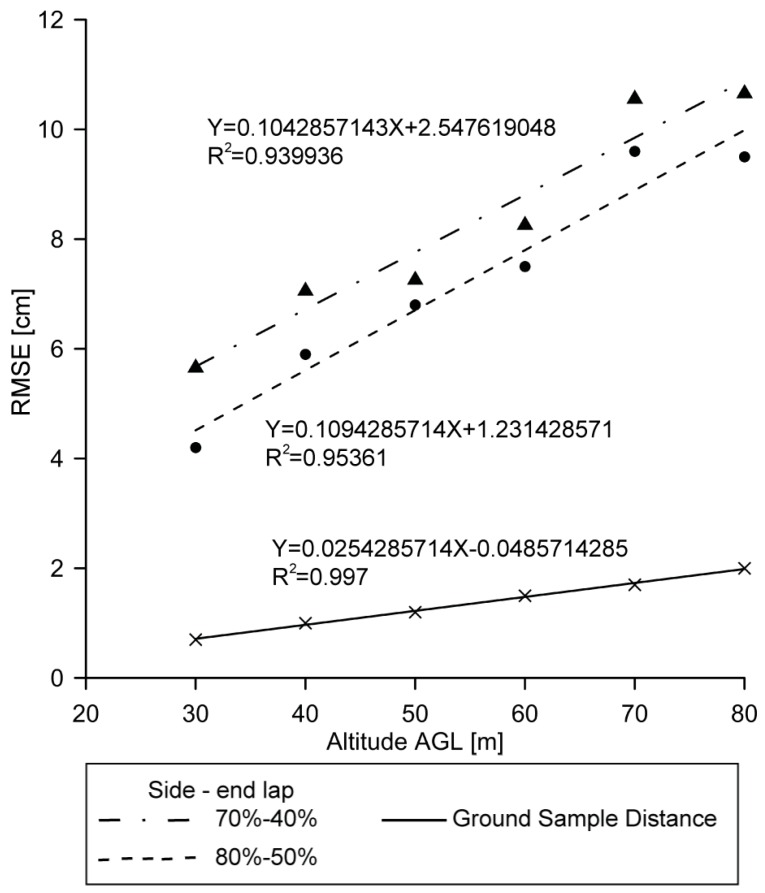
Linear model analyzing forward and side lap settings against altitude AGL and RMSE.

Figure 9 shows the forward and side overlap settings related to altitude AGL and RMSE in the flights where GCPs were used. Altitude AGL and RMSE show a linear relationship with a correlation coefficient higher than 0.9, independently of forward and side lap settings. The two linear models represented tend to converge. At lower altitudes AGL, the distance between both adjusted lines is greater while tending to converge as altitudes AGL increases. The higher forward and side overlap settings correlate with a lower RMSE having more influence on RMSE at lower altitudes AGL. On the other hand, Figure 9 also shows that altitude AGL has more impact on RMSE than forward and side overlap settings.

Also in Figure 9, GSD is represented by a continuous line, which has a moderate slope compared to the linear models of error, representing its correlation to RMSE. The mean ratio between RMSE and GSD of all UAV flights in this study was 5, suggesting that the expected RMSE of an UAV orthomosaic is five times greater than GSD of images. Ref. [41] obtained a ratio of 3.7 in an experiment on an archaeological site. This difference between results may be due to the fact that they used circular targets as their well-defined check points, while, in this study, in order to approximate an applied assessment, elements of archaeological interest were used as intersection and corner check points that are more diffuse and therefore more difficult to locate and measure.

### 3.2. Assesment of Relative Positional Accuracy

Referring to relative positional accuracy (Table 4), RHSD was stable when GCPs were used to define the coordinate reference system in the aerial triangulation phase, ranging from 4.5 cm to 6.5 cm, increasing as altitudes AGL increased and with both forward and side lap settings. RHSD showed lower values with the higher forward and side lap setting.

Without GCPs, RHSD showed random behavior similar to the results obtained in the absolute positional accuracy assessment above. As such, the orthomosaics obtained only using data from the UAV navigation system were rotated, translated and scaled respecting the coordinate reference system. These products were not useful even in a relative coordinate system because any linear or surface measurement is not going to appear correct because the coordinate system was not well defined.

Therefore, designing a UAV flight plan requires defined technical specifications related to illumination, resolution and spatial quality. These parameters have to be considered equally to produce an adequate UAV orthomosaic. Illumination and material effects e.g., marble and image saturation, define the time frames for flying, which is important if the time frames are narrow and flights have to be short. In regards to spatial accuracy, the expected RMSE is five times greater than the GSD registered on flight. AGL is the parameter that mainly influences RMSE. While using higher forward and side overlap settings guarantee greater positional accuracy, flight duration will be increased. Finally, if the navigation system of the UAV is inaccurate, it is necessary to use GCPs, even if the orthomosaics are going to be used in a relative coordinate system.

In this manuscript, spatial resolution of UAV orthomosaicks has been assessed to survey archaelogical areas. Another useful geomatic product in archaeology is model digital surface (MDS). These models can be generated using passive sensors, as we explain and use in this manuscript, or by active sensors like LiDAR. LiDAR sensors can be mounted on manned platforms, airborne and terrestrial [42] or unmanned platforms [43], and have been used successfully in archaelogical prospection [44]. Future works should be performed to compare MDS obtained by LiDAR and aerial imagery and assessing spatial resolution of LiDAR sensor onboard UAV and the influence of flight parameters.

## 4. Conclusions

This study has shown that UAV systems are useful complements for archaeological mapping, such as GNSS measurements or aerial photogrammetry among others. The main objective of this investigation was to analyze the configuration and technical specifications of a multi-rotor UAV equipped with a RGB sensor to produce accurate orthomosaics to be applied in archaeological applications.

Concerning spatial resolution, flight altitude AGL is an important parameter because of the degree detail achieved in the orthomosaic image to be used to for analysis and study in an archaeological context. Additionally, adequate values of altitude AGL and forward and side overlap settings have to be applied because of their impact on flight duration and positional accuracy of absolute and relative RMSE obtained. Our results have shown a ratio between RMSE and GSD of UAV flights equal to 5. Whenever possible, higher percentages of forward and side overlap are recommended for UAV flights. Other flight planning configurations, including transversal laps, can be carried out in future works to study their impact on flight duration and accuracy of results. Moreover, if the UAV’s navigation system is not accurate enough, GCPs can be used instead, recalling that even if working in a relative coordinate reference system, any linear or superficial measurement is not going to be accurate without GCPs. The use of navigation systems based on differential-GPS can be an alternative to GPC measurements to be taken into account, assessing its influence in spatial resolution.

The results herein presented can be used to configure flight missions using a RGB sensor onboard a multi-rotor UAV to maximize the spatial positional accuracy of orthomosaics to be used in archaeological mapping.

## Figures and Tables

**Table 1:** Results of the field-calibrated Sony NEX-7 (Sony Corporation, Minato, Tokyo, Japan).

Parameters	Value	Parameters	Value
Focal length (mm)	16.6286	Radial Distortion K2	0.0290259
Principal point—X (mm)	12.2712	Radial Distortion K3	−0.0338008
Principal point—Y (mm)	7.76064	Tangential Distortion T1	−0.00162188
Radial Distortion K1	−0.0108059	Tangential Distortion T2	−0.00094999

**Table 2:** Flight durations and the number of images taken at different altitudes Above Ground Level (AGL) and forward and side lap settings.

Altitude AGL (m)	Forward/Side Lap 70%–40%		Forward/Side Lap 80%–50%	
	Time Duration	Num Images	Time Duration	Num Images
30	0:07:12	27	0:07:35	34
40	0:02:40	12	0:06:03	27
50	0:02:12	10	0:03:08	14
60	0:01:52	8	0:02:44	12
70	0:00:46	4	0:00:56	5
80	0:00:33	3	0:00:56	5

**Table 3:** Absolute positional accuracy results factoring altitude AGL, percentage of forward and side overlap and processing with or without GCPs.

Altitude AGL (m)	GSD (cm)	Forward/End Lap (%)	GCP RMSE (cm)	No GCP RMSE (cm)
30	0.7	80%/50%	3.8	507.8
		70%/40%	5.4	178.0
40	1	80%/50%	5.9	138.4
		70%/40%	6.3	73.3
50	1.2	80%/50%	6.2	99.2
		70%/40%	6.5	53.8
60	1.5	80%/50%	6.9	120.7
		70%/40%	6.8	151.9
70	1.7	80%/50%	9.6	179.6
		70%/40%	9.2	229.0
80	2	80%/50%	9.5	179.6
		70%/40%	10.0	934.2

AGL: Above Ground Level, GSD: Ground Sample Distance, GCP: Ground Control Point, RMSE: Root Mean Square Error.

**Table 4:** Relative horizontal spatial deviation.

GCPs	Forward–End Lap	Altitude AGL (m)					
		30	40	50	60	70	80
With	70%–40%	5.1	5.5	6.1	6.2	6.2	6.5
	80%–50%	4.5	4.5	4.9	5.0	5.3	5.1
Without	70%–40%	44.0	63.3	56.7	38.1	44.4	88.8
	80%–50%	21.4	33.4	19.7	6.9	42.5	16.6
